# The relationship between genotype, psychiatric symptoms and quality of life in adult patients with sickle cell disease in São Paulo, Brazil: a cross-sectional study: A relação entre genótipo, sintomas psiquiátricos e qualidade de vida em pacientes adultos com doença falciforme em São Paulo, Brasil: um estudo transversal

**DOI:** 10.1590/1516-3180.2015.00171105

**Published:** 2015-08-21

**Authors:** Érika Bergamini Mastandréa, Fátima Lucchesi, Marcela Mayumi Gomes Kitayama, Maria Stella Figueiredo, Vanessa de Albuquerque Citero

**Affiliations:** I Escola Paulista de Medicina Universidade Federal de São Paulo Department of Psychiatry São Paulo SP Brazil MSc. Psychologist, Department of Psychiatry, Escola Paulista de Medicina (EPM), São Paulo (SP), Brazil; II Universidade Federal de São Paulo Universidade Federal de São Paulo Escola Paulista de Medicina Department of Clinical and Experimental Oncology São Paulo SP Brazil MD. Hematologist, Department of Clinical and Experimental Oncology, Escola Paulista de Medicina (EPM), Universidade Federal de São Paulo (Unifesp), São Paulo (SP), Brazil; III Escola Paulista de Medicina Universidade Federal de São Paulo Department of Psychiatry São Paulo SP Brazil PhD. Psychiatrist, Department of Psychiatry, Escola Paulista de Medicina (EPM), São Paulo (SP), Brazil

**Keywords:** Anemia, sickle cell, Quality of life, Anxiety, Depression, Alcoholism, Anemia falciforme, Qualidade de vida, Ansiedade, Depressão, Alcoolismo

## Abstract

**CONTEXT AND OBJECTIVE:**

Health-related quality of life (HRQoL) may be worsened in sickle cell patients due to the presence of psychiatric disorders. The aims of this study were to describe the psychiatric symptoms in Brazilian sickle cell patients and to evaluate the relationship of these symptoms to the genotype of the disease and the subject's HRQoL.

**DESIGN AND SETTING:**

Cross-sectional study conducted at the hematology outpatient clinic, Hospital São Paulo.

**METHODS:**

Adult patients with sickle cell disease completed the Medical Outcome Study - Short Form 36 and the Patients' Health Questionnaire. Clinical data were gathered from their medical files. Linear regression models were developed to study the dependency of HRQoL domains on the genotype controlling for psychiatric symptoms.

**RESULTS:**

In the study period, 110 patients were evaluated. The most frequent psychiatric symptom was depression (30%), followed by anxiety (12.7%) and alcohol abuse (9.1%). Patients with the more severe genotype (SS and Sβthal^0^) showed lower scores for the "general health" and "role-physical" HRQoL domains, without interference from psychiatric symptoms. In the "role-physical" domain, the more severe genotype operated as a protective factor for HRQoL (β = 0.255; P = 0.007).

**CONCLUSION:**

The more severe genotypes worsened HRQoL in two domains of physical health (general health and role-physical), but they did not have any influence on mental health, thus suggesting that physicians should be more attentive to aspects of HRQoL relating to the functionality of sickle cell disease patients, so as to be aware of the limitations that these patient live with.

## Abstract (pt)

**CONTEXTO E OBJETIVO:**

A qualidade de vida relacionada à saúde (QVRS) pode ser piorada em pacientes com doença falciforme na presença de transtornos psiquiátricos. O objetivo deste estudo é descrever a sintomatologia psiquiátrica presente no paciente brasileiro com doença falciforme e avaliar a relação desses sintomas com o genótipo da doença e a QVRS do sujeito.

**TIPO DE ESTUDO E LOCAL:**

Estudo com delineamento transversal, realizado no ambulatório de Hematologia do Hospital São Paulo.

**MÉTODOS:**

Adultos com doença falciforme responderam ao Questionário de Qualidade de Vida (SF-36) e ao Questionário sobre a Saúde do Paciente (PHQ). Dados clínicos foram obtidos no prontuário médico. Modelos de regressão linear foram desenvolvidos para estudar a dependência dos domínios de QVRS no genótipo com controle para sintomas psiquiátricos.

**RESULTADOS:**

No período do estudo, 110 pacientes foram avaliados. O sintoma psiquiátrico mais frequente foi o depressivo (30%), seguido do ansioso (12,7%) e de abuso de álcool (9,1%). Os pacientes com genótipo mais grave (SS e S βthal^0^) apresentaram menores médias nos domínios de QVRS de "saúde geral" e de "aspectos físicos", sem interferência dos sintomas psiquiátricos. No domínio "aspectos físicos", o genótipo mais grave funcionou como fator protetor da QVRS (β = 0,255; P = 0,007).

**CONCLUSÃO:**

Os genótipos mais graves pioraram a QVRS em dois domínios do componente físico ("aspectos físicos" e "saúde geral"), mas não influenciaram o componente mental, sugerindo que o médico deve estar atento aos aspectos da QVRS relacionados com a funcionalidade do portador da doença falciforme, conhecendo as limitações com as quais o paciente convive.

## INTRODUCTION

Sickle cell disease is associated with high rates of morbidity and mortality among the individuals affected.^1^ Data from other countries have shown that lack of psychiatric care decreases the patient's quality of life while increasing healthcare costs.^2^^,^ 3 Pain crises, hospitalization, blood transfusions and other morbidities have been found to have a significant impact on these patients' quality of life.^4^ The disease also influences these individuals' work capacity: they generally have low income levels and little access to healthcare services, which in turn has a significant impact on quality of life.^5^ Functional status and quality of life may be worse among adult patients with sickle cell disease due to the presence of comorbitities,^2^ taking into account that psychiatric patients suffer greater harm in terms of quality of life than do patients with no psychiatric comorbidities.6 In Brazil, it has become a public health issue because it leads to major socioeconomic problems due to the diminished job opportunities that these patients face in the labor market.^7^

A review study found that, in comparison with other chronic diseases, adults and children with sickle cell disease present significantly diminished quality of life.^8^ This effect is also related to the way in which people cope with the disease, the high symptom burden, sufferers' housing conditions (rural or urban), their professions, their genotype and the use of opioids.^6^

The PiSCES project (Pain in Sickle Cell Epidemiology Study) showed that Afro-American patients with sickle cell disease presented severely impaired lives and that depression was a powerful predictor of the component scores for the physical and mental aspects of quality of life, rather than the genotype. The presence of anxiety symptoms had the same impact as the presence of depression on pain and quality of life. The higher the patient's anxiety was, the more days he or she experienced pain, with or without pain crises.^9^ Patients with sickle cell disease showed lower quality of life in mental component scores, compared with patients with cystic fibrosis, as well as lower scores in the "bodily pain," "vitality" and "social functioning" domains, in comparison with asthma patients.^3^ Compared with patients undergoing dialysis, sickle cell patients also showed less "bodily pain," "general health" and "vitality".^3^ Sickle cell disease and its complications lead to a major burden for patients, especially the impact of the pain and the symptoms that they have to endure on their daily routine and social relationships. Often, there is an increase in psychological morbidity, such as depression and the ways of coping with it.^10^

Acute pain is a characteristic of sickle cell disease and requires frequent hospitalization, which affects the patients' quality of life. In this regard, a longitudinal study was carried out on 510 subjects through self-assessment of mood, pain, health and quality of life upon hospital admission, prior to discharge and then again one week after discharge. As a result, it was found that better self-assessment of health, general mood and quality of life led to a significant improvement in pain reduction for patients with sickle cell disease upon hospital admission, prior to discharge and after one week of follow-ups.^11^

In Brazil, no such data have been analyzed. Understanding the relationship between the presence of psychiatric symptoms, clinical characteristics of the disease and quality of life will make it possible for appropriate interventions to be developed in order to improve the quality of life of Brazilians with sickle cell disease. 

## OBJECTIVE

The objective of this study was to evaluate the relationship between the quality of life of Brazilian sickle cell patients, the genotype of the disease and the presence of psychiatric symptoms. Specifically, the study aimed to evaluate the role of genotype on sickle cell patients' quality of life. Taking the scientific literature into account, it was hypothesized that the presence of psychiatric symptoms would decrease the quality-of-life score but the genotype would not have any relationship with this.

## METHOD

This research project was approved by the Research Ethics Committee of Universidade Federal de São Paulo (procedural no. 005/08). A cross-sectional study was concluded. All patients who were being treated at the hematology outpatient unit at the university were invited to participate, and the study was conducted from November 2009 to July 2011. All patients who were aged 18 years or older, presented preserved cognitive capability and were assessed by means of the Mini-Mental State Examination were included in the study. The exclusion criterion determined that patients complaining of pain unrelated to sickle cell disease could not participate. The sample size was determined considering the prevalence of the event of interest. Given the study hypothesis, the presence of psychiatric symptoms was the event of interest and, using the PiSCES project as the reference, the prevalence of these symptoms was between 27% and 31% (depressive symptoms and alcohol abuse, respectively). Therefore, to ensure that the sample would include at least 30 patients with psychiatric symptoms, 100 patients with sickle cell disease would be needed. We added another 10% to this sample size to allow for losses, thus resulting in a final sample of 110 patients. Patients were approached on the days of their medical appointments.^12^^,^ 13

The patients were contacted (during their routine appointments) and asked to give their consent to take part in the study and to schedule a first interview at the health center. At these appointments, one research field coordinator and two interviewers were always present at the outpatient unit. After agreeing to take part in the study, the patients reviewed and signed the informed consent form. Each patient was then interviewed and provided the baseline measurements for the study, which comprised sociodemographic information (such as gender, age, marital status, level of education and family income) and psychiatric information. Other data (for example, SS, Sβthal^0^, SC and Sβthal^+ ^genotypes and clinical complications of sickle cell disease) were taken from the patient's medical files. For analysis purpose, the genotype was considered to be a dichotomized variable in which the SS and Sβthal^0^ genotypes were grouped as "genotypes with a more severe clinical form" (or simply "more severe genotypes"), and the SC and Sβthal^+ ^genotypes were grouped as "genotypes with a less severe clinical form" (or simply "less severe genotypes"). The clinical complications of sickle cell disease were not used as a covariable in the analysis models because they were a rare event.

In order to evaluate quality of life, the Medical Outcome Study Short Form 36 was used,^14^ given that this is a generic instrument for assessing quality of life that has already been validated in Brazil.^15^ It comprises 36 items distributed into eight domains: physical functioning, role-physical, bodily pain, general heath, vitality, social functioning, role-emotional and mental health. The first four domains address the physical component and the other domains focus on the mental component of quality of life. This instrument assesses negative aspects of health (such as diseases and illnesses) and also positive aspects relating to wellbeing.^16^ Each domain generates a score ranging from 0 to 100%, and the higher the score is, the better the quality of life that patients are considered to have in that specific health sector. 

In order to assess the presence of symptoms of anxiety, depression and alcohol abuse, the Patient Health Questionnaire (PHQ) screening tool was used.^17^ The Brazilian version obtained by Pfizer (a pharmaceutical company that contributed to the development of the PHQ instrument) was applied. The PHQ-9 (the part of PHQ that explores depressive symptoms) has also been validated in Brazil.^18^ The depression subscale has 9 questions, which together may result in a score from 0 to 27 points, meaning that patients who score from 0 to 9 have no symptoms and those who score 10 and above present symptoms of depression. The anxiety subscale has 7 questions, which together may result in a score ranging from 0 to 21 points; it also has a cutoff point of 9/10. The alcohol abuse subscale has 5 items, which together result in a score from 0 to 5; a patient who scores 1 or more already meets the criterion for alcohol abuse.

A description of the sample and its relationship with the sociodemographic and clinical characteristics was analyzed. Quality of life was described using means and standard deviations, and was then compared according to gender, genotype, presence of depression and anxiety symptoms and presence of alcohol abuse, always using Student's *t*-test for two independent samples. In order to establish the predictive value that the genotype has in relation to quality of life, we proposed to develop eight linear regression models in which the dependent variable in each model would be one quality-of-life domain score that had been affected by the genotype in the association study. The independent variable was the more severe/less severe genotype and the control variables were the presence of psychiatric symptoms and patient gender. Results with P < 0.05 were considered statistically significant. The SPSS 20.0 software was used.

## RESULTS

None of the patients were undergoing psychological treatment at the time of the interview. In the study period, out of the 133 patients who had been invited, 110 were accepted and took part in the study (9 did not meet the inclusion criteria and 14 refused to participate). Comparison between all the 110 individuals included and the 14 who refused to participate in the study did not show any statistically significant differences regarding gender, age or genotype.

The 110 patients included were predominantly women (61.8%), had an average age of 30 years (standard deviation = 11 years, ranging from 18 to 67 years), were mostly single (70%) and had an average annual income of US$ 19,500 (ranging from U$ 3,000 to U$ 60,000). Eighty patients (72.7%) had the more severe genotypes for sickle cell disease. The presence of depression symptoms was found in 30% of the patients, considering that the median symptom score was 6, ranging from 0 to 27. Anxiety symptoms were present in 12.7%, with a median score of 0, ranging from 0 to 14. Alcohol abuse symptoms were found to be present in 9.1%, also with a median score of 0, and ranging from 0 to 4. 

Table 1 shows the distribution of quality-of-life domain scores based on the presence of psychiatric symptoms, genotype and gender. Female patients with no psychiatric symptoms and less severe genotypes had higher scores for quality of life than other patients. Depression symptoms affected the quality of life of patients with sickle cell disease in all domains scores except "general health." The presence of anxiety symptoms was also not connected with the "general health" domain score and did not affect the "role-physical" domain score. In relation to alcohol abuse, it was found that this problem was not associated with any quality-of-life domain scores. 

**Table 1.** Distribution of the quality-of-life domain scores (mean ± standard deviation) in relation to the presence or absence of depression or anxiety symptoms, alcohol abuse, genotype and gender

	Depression symptoms		Anxiety symptoms		Alcohol abuse		Genotype		Gender	
	Present versus absent	P	Present versus absent	P	Present versus absent	P	More severe versus less severe	P	Male versus female	P
Physical functioning	70.4 ± 15.6 versus 79.6 ± 15.1	0.01	67.4 ± 16 versus 77.8 ± 67.4	0.03	78.1 ± 12.6 versus 76.4 ± 16.3	0.76	75.3 ± 15.2 versus 80.8 ± 16.9	0.12	78.3 ± 19 versus 75.4 ± 13.8	0.37
Role-physical	70.8 ± 19.4 versus 80.1 ± 20.6	0.01	71.4 ± 21.6 versus 78.1 ± 71.4	0.25	77.5 ± 21.8 versus 77.2 ± 20.5	0.97	73.7 ± 19.8 versus 87 ± 19.6	0.00	77.3 ± 22.4 versus 76.8 ±19.5	0.89
Bodily pain	58.5 ± 21.8 versus 67.9 ± 22.8	0.03	52.4 ± 24.4 versus 66. 2 ± 52.4	0.05	59.1 ± 27.6 versus 65.1 ± 22.1	0.43	63.1 ± 22.3 versus 70.5 ± 23.5	0.15	71.3 ± 25.1 versus 61.1 ± 20.6	0.03
General health	49.9 ± 16.4 versus 56.9 ± 18.2	0.06	48.4 ± 17.7 versus 55.8 ± 48.4	0.17	46.5 ± 15.7 versus 55.8 ± 18	0.12	51.6 ± 17.1 versus 63.6 ± 17.3	0.00	51.5 ± 19 versus 56.7 ± 17.1	0.15
Vitality	54.2 ± 17.8 versus 67.3 ± 14	0.04	51.9 ± 17.8 versus 64.7 ± 51.9	0.01	65.8 ± 12 versus 62.9 ± 16.7	0.59	62.9 ± 16.1 versus 64.5 ± 17.1	0.67	65.7 ± 17.8 versus 61.8 ± 15.2	0.23
Social functioning	61.8 ± 21.3 versus 82.2 ± 17.9	0.00	60 ± 27.3 versus 77.7 ± 60	0.01	68 ± 25.7 versus 76.4 ± 20.4	0.23	73.8 ± 19.9 versus 82 ± 22.7	0.07	77 ± 24 versus 75.2 ± 19.1	0.68
Role-emotional	66.1 ± 21 versus 87.72 ± 17.9	0.00	65.4 ± 21.1 versus 83.8 ± 65.4	0.00	71.6 ± 22.2 versus 82.4 ± 21	0.13	79.3 ± 21.7 versus 87.3 ± 19.2	0.08	81.3 ± 22.1 versus 81.3 ± 20.8	0.99
Mental health	53.9 ± 16.7 versus 74.1 ± 14.3	0.00	51 ± 16.3 versus 70.4 ± 51	0.00	59.6 ± 16.5 versus 68.9 ± 17.7	0.12	65.9 ± 17.9 versus 73.9 ± 16.1	0.04	71.4 ± 18.6 versus 66.1 ± 16.9	0.14

As seen in Table 1, a patient's genotype only influenced quality-of-life domain scores in terms of "role-physical," "general health" and "mental health." A patient's gender and the presence of alcohol abuse were not associated with any of these domain scores. Therefore, these two control variables were not used in regression models.

The "general health" domain score for quality of life showed lower scores when patients had a more severe genotype, and did not show any connection with anxiety or depression symptoms, so much that the regression study controlled by these variables was unnecessary for showing the influence of genotype on "general health." Therefore, two regression models were developed (Table 2), to test the independence of the genotype as a predictive factor for the "role-physical" domain score controlled for depression and anxiety symptoms (Model 1); and the "mental health" domain score, controlled for anxiety and depression symptoms (Model 2). Model 1 showed that the presence of a less severe genotype was a predictor of better quality of life in "role-physical," operating as a protective factor, with an explanatory value for the variation of "role-physical" of 10.3%. Model 2 showed that the presence of a less severe genotype was not a predictor of quality of life in "mental health," after it was controlled for psychiatric symptoms. In other words, the presence of anxiety and depression symptoms is what generated the variation of the "mental health" domain score, not the genotype. 

**Table 2.** Evaluation of genotype as a predictor of mental health, with depression and anxiety symptom scores as the control variables

		Beta	t	P	95% confidence interval	
					Lower limit	Upper limit
Model 1 (role-physical)	Depression score	-0.200	-2.16	0.033	-1.266	-0.055
	Genotype	0.255	2.76	0.007	3.301	20.178
Model 2 (mental health)	Depression score	-0.621	-8.19	0.000	-2.195	-1.339
	Anxiety score	-0.407	-4.52	0.000	-2.802	-1.094
	Genotype	0.131	1.46	0.148	-1.879	12.292

## DISCUSSION

In this study, the more severe genotypes (SS and Sβthal0) decreased the quality of life in two physical domains (role-physical and general heath), but they did not interfere with mental functioning. In the study by Panepinto,^19^ the laboratory markers for sickle cell disease, such as genotype, fetal hemoglobin and lactate dehydrogenase were correlated with deteriorated quality of life. According to van Tuijin et al.,^20^ the genotype and the presence of chronic injury in organs were not significantly related to quality of life, which was worsened by the pain level, profession and level of education, similarly to the study by Pereira et al.^4^

The previous results were similar to those of McClish et al.,^3^ who did not find any correlation between the genotype and quality of life. However, Sogutlu et al.^21^ showed that the Sβthal^0^ genotype was related to greater numbers of pain crises. In the population studied, the more severe genotype (SS, Sβthal^0^) was the one that proved to be predictive of better quality of life and operated as a protective factor in relation to the role-physical domain. Based on the results from studies on genotypes and quality of life, it is important to raise another hypothesis, stating that such results may be related to the quality of treatment that patients are given, i.e. whether the treatment has been managed appropriately by the physician, whether it has been followed correctly by the patient and whether the patient can afford to buy the medication. 

Having appeared in the study with a high frequency (80%), the more severe genotypes and all of the quality-of-life domains except for "general health" are closely related to depression symptoms. Anxiety symptoms were also related to quality of life except for the "role-physical" and "general health" domains. The study by Asnani et al,^10^ showed that the complications endured by patients with sickle cell disease resulted in increases in psychiatric complications, especially depression, and in the precarious ways in which patients cope with it. This was similar to the findings of the study by Ameringer et al.,^22^ which also included sleep disorders, anxiety and stress. For physical relief, Panepinto and Bonner reported that hydroxyurea was used.^6^ This brought a significant improvement in patients' quality of life, in comparison with patients taking a placebo. In a study by Sogutlu et al.,^21^ on physical symptoms and pain, the quality of life of patients with sickle cell disease decreased and anxiety and depression were present. Based on the considerations mentioned above, we can consider that treating a patient with sickle cell disease in a more comprehensive way is fundamental to improving his or her quality of life. Raising awareness about what the disease means and how to deal with its consequences may be fundamental factors for improving quality of life. Availability to attend treatment or to go to a collection site may be obstacles. 

In this study, quality of life was not shown to decrease through the presence of alcohol abuse (9.1%). In comparison with the PiSCES study, which was conducted using Afro-American samples,^23^ 31.4% of the patients with sickle cell disease were alcohol abusers, i.e. they showed prevalence of abuse that was much higher than here in Brazil. Quality of life was very similar between the two groups (abusers and non-abusers), except that unexpectedly, alcohol abusers had a better general score for "role-physical." Apart from the possible function of body hydration, and even though not recommended, use of alcohol may also have led to a soothing effect with the first few doses, thereby providing a sensation of immediate relief, although this feeling disappeared as consumption increased. 

Likewise, as described in a study by Citero et al.,^24^ in relation to catastrophic thinking, it is not possible to compare the mental adaptation that an individual with sickle cell disease has to endure with that of another individual with a non-fatal disease who has not had complications present since childhood. A study by Adams-Graves et al.^25^ showed that there were significant differences in quality-of-life scores among patients with sickle cell disease and among patients with rheumatoid arthritis, when an analysis was carried out in terms of gender, family income, level of education and comorbidities. Although it can be seen that chronic diseases affect quality of life significantly, generalizations stating that the interference caused by sickle cell disease is greater than that from other diseases cannot be made. 

The sample for this study comprised 110 patients, among whom 80% had the more severe genotype. This was similar to what was found in studies conducted by Mann-Jiles^26^ and by Adams-Graves et al.^25^

### Clinical implications 

Physicians need to be more attentive to quality-of-life characteristics, which may be deteriorating. The "general health" domain accounts for issues relating to whether patients believe that they are healthy or that they are likely to get sick. The "role-physical" domain assesses whether the patient has had any trouble with regular activities within the last four weeks due to physical health issues, such as difficulties or constraints. In this case, the physician should ask patients about their ability to work and take part in leisure activities, and how they feel about their health in general. This is important in helping the physician find out whether patients' daily routines are being affected. In other words, challenging the functionality of patients with sickle cell disease will help the physician learn about the limits that these patients have to deal with.

### Limiting factors of this study

One of the limiting factors that should be taken into account relates to the lack of pain records for these patients. Based on the literature analyzed, pain is also a predictor of quality of life for sickle cell disease patients. In the various studies that we made use of in preparing this manuscript, the pain aspect was seen countless times, but we have not addressed this topic in greater depth because it is part of a longitudinal study, which has not yet been concluded. Another limiting issue relates to the composition of the sample, since it was made up of patients who were receiving assistance at a university center of excellence and therefore were in a privileged position in relation to other patients treated at regular health centers by general practitioners who do not have specific training in sickle cell disease. The university healthcare service may also offer more medication options and have higher quality in the assistance provided. Another problem is that the subjects were from a convenience sample, which means that there is a possibility of bias in that patients who agreed to participate in the study may have been subjects with better outcomes from the treatment, and consequently higher quality of life.

## CONCLUSIONS

The more severe genotypes worsened the health related quality of life in two domains of physical health (general health and role-physical), but they did not have any influence on mental health, thus suggesting that physicians should be more attentive to aspects of quality of life relating to the functionality of sickle cell disease patients, so as to be aware of the limitations that these patients live with.
